# Modeling and Mapping of Combined Noise Annoyance for Aircraft and Road Traffic Based on a Partial Loudness Model

**DOI:** 10.3390/ijerph18168724

**Published:** 2021-08-18

**Authors:** Wonhee Lee, Chanil Chun, Dongwook Kim, Soogab Lee

**Affiliations:** 1 Department of Aerospace Engineering, Seoul National University, 1 Gwanak-ro, Gwanak-gu, Seoul 08826, Korea; worry@snu.ac.kr (W.L.); ehdrk1029@snu.ac.kr (D.K.); 2 Environment & Safety Research Center, Samsung Electronics, Gyeonggi-do, Hwaseong-si 18448, Korea; chanil.chun@samsung.com

**Keywords:** annoyance model, annoyance map, aircraft noise, road traffic noise, short-term annoyance, long-term annoyance, background noise effect

## Abstract

Complex transportation systems often produce combined exposure to aircraft and road noise. Depending on the noise source, the annoyance response is different, and a masking effect occurs between the noise sources within the combined noise. Considering these characteristics, partial loudness was adopted to evaluate noise annoyance. First, a partial loudness model incorporating binaural inhibition was proposed and validated. Second, short- and long-term annoyance models were developed using partial loudness. Finally, the annoyance of combined noise was visualized as a map. These models can evaluate the annoyance by considering both the intensity and frequency characteristics of the noise. In addition, it is possible to quantify the masking effect that occurs between noise sources. Combined noise annoyance maps depict the degree of annoyance of residents and show the background noise effect, which is not seen on general noise maps.

## 1. Introduction

Awareness of the importance of environmental pollution, an inevitable issue, is gradually spreading. Noise, an environmental pollutant, is an unwanted sound that can affect human health [1]. In addition, unlike other environmental pollutants, noise pollution continues increasing due to industrialization and traffic growth [2]. Increasing noise exposure from multiple sources causes widespread annoyance. Consequently, noise sources and levels are regulated in several countries [3,4]. Many researchers have studied the effects of noise exposure [5,6,7], which causes auditory (e.g., hearing loss) and non-auditory (e.g., sleep disturbance, hypertension, and cardiovascular diseases) health effects. Furthermore, the most significant impact of noise exposure in many countries is the perception of annoyance [8,9].

Past noise exposure largely stemmed from a single event. The previous studies mentioned above considered single noises or treated combined noises as the sum of single noise sources. However, the development of transportation systems has increased noise exposure from combined sources. For example, residents living near airports are exposed to aircraft and road traffic noises. Therefore, multiple studies have been conducted to quantify the annoyance of multisource noise [10,11,12,13,14,15]. Brink et al. explored the masking effects of road traffic noise on aircraft noise annoyance [10]. Hong et al. compared the annoyance of single and combined noise through annoyance evaluation according to the conditions of noise exposure [11]. Wothge et al. studied the annoyance of the combined noise of aircraft and road traffic noise or the combined noise of aircraft and railway noise and showed that noise annoyance is determined by the sound source judged to be more annoying [12]. Lechner et al. showed the cumulative effect of each source of combined noise in the annoyance response through the survey data [13]. The dose-response model for combined noise annoyance, suggested by Miedema [14], is derived using the method from Vos, called the annoyance equivalent model [15]. The Miedema model applies the annoyance-equivalent factor from a reference source [14].

Meanwhile, along with research on annoyance modeling, studies on visualizing annoyance as noise maps (first suggested by Knauss) have also been conducted [16]. The annoyance potentials were calculated using the annoyance map depending on the source type [17]. The annoyance map of road traffic noise was drawn with survey data on the percentage of highly annoyed individuals [18]. Another study found a moderate correlation between noise annoyance and exposure levels to road traffic noise [19]. In addition, research on 3D noise maps has been actively conducted due to advances in technology [20]. Along with advances in computer graphics and virtual reality technologies, several studies have been conducted to explore the noise of virtual reality. Law et al. implemented realistic 3D noise models using real building photos and noise models and used them interactively, allowing users to experience them in a virtual reality environment [21]. Berger et al. combined data visualization and sonification technology to enable users to explore and hear road traffic noise in an immersive real-time urban environment [22].

This study focused on two points in evaluating the annoyance of combined noise. The first is that aircraft noise is perceived to be more annoying than road noise at the same noise level [11,12,13]. It makes modeling difficult for quantifying annoyance with physical noise indices such as the day-night noise level. Loudness, a psychoacoustic factor, was adopted to improve in this study. Loudness is a sensation regarding a category of noise intensity [23]. Since loudness can consider both the intensity and frequency characteristics of the noise, it can better reflect the noise magnitude than the physical indicators. The second point is a masking effect or background noise effect between each noise source within the combined noise [10]. The masking effect is defined as the interference with the perception of one sound (the target) by another sound (the masker) [24]. Often the masker sound does not entirely mask the target sound, but merely reduces its loudness, thereby partially masking.

In this work, the concept of partial loudness was adopted to quantify the masking effects present in combined noise. Partial loudness means the loudness of each noise source considering the masking effect in the presence of multiple noise sources [25]. The masking effect can be quantified because it calculates the partial loudness according to the target and masker sound. The same concept was used in our previous study as well [26]. However, research on the annoyance of residents has not been conducted. In addition, binaural inhibition was not considered in the calculation of loudness. Binaural inhibition is the concept that the sound heard from one ear reduces the internal response of the signal at the other ear. Kim et al. executed the laboratory test to compare monaural and binaural listening to show that annoyance from the single and dummy head results in different tendencies with increasing noise level yet not as twice [27]. For this reason, in our previous study, a dummy head was used for binaural recording [26]. However, loudness was calculated with a model based on the assumption that loudness is summed across the ear [28]. Moore et al. developed an improved loudness model by adding binaural inhibition functions [29]. It was applied in this study.

Therefore, this study proposes combined noise annoyance models from aircraft and road traffic noises. Since these models were calculated using loudness, they include intensity factors and frequency characteristics. By adopting the partial loudness concept, it was possible to quantify the effect of background noise between combined noise sources. In addition, we improved the accuracy in calculating the loudness by considering the binaural effect, which was not considered in our previous study. The partial loudness model considering binaural inhibition was developed and validated using a listening test to evaluate loudness of the combined noise of aircraft and road traffic noise. Based on the improved partial loudness model, the two types of models presented herein depend on noise exposure. The first is a short-term annoyance model derived by logistic regression analysis of the results obtained from the annoyance response experiment for each noise. The second model is a long-term annoyance model for residents exposed to noise for more than a year. The long-term model was derived using a noise map consisting of resident survey data and aircraft and road traffic noises in the research area. Additionally, this study implements an annoyance map based on a long-term model to visualize the effect of annoyance on noise exposure. Because it was based on partial loudness, it was possible to display aircraft and road noise separately from main and background noise. Therefore, the masking effect or the background noise effect was considered, and we demonstrate the relationship between the combined noise sources.

## 2. Materials and Methods

### 2.1. Partial Loudness Calculation

Currently, loudness models are designated as ISO standards, and there are two models. Zwicker’s model specifies two methods for estimating the loudness and loudness level of sounds as perceived [30]. The first method is intended for stationary sounds, and the second method for time-varying sounds. Moore’s model specifies a method for estimating the loudness and loudness level of stationary sounds as perceived [31]. Since preference cannot be specified for either of these two models, it is up to the user to choose the method that appears most appropriate for the given situation. In this research, a partial loudness model based on Moore’s model was adopted to calculate the magnitude of the combined noise.

The partial loudness model is an advanced model that calculates the partial loudness of combined noises with each noise source [25,28,29,32]. This model calculates loudness by classifying each noise source into target and background noises in case of multiple noises. It is assumed that the total loudness of combined noise is composed of the sum of the partial loudness of each noise source.

(1) $$\begin{array}{c} N_{TOT}^{{}^{\prime }}=N_{Target}^{{}^{\prime }}+N_{Background}^{{}^{\prime }} \end{array}$$

where, $N_{TOT}^{{}^{\prime }}$ is total loudness of combined noise. $N_{Target}^{{}^{\prime }}$ is partial loudness of the target noise and $N_{Background}^{{}^{\prime }}$ is partial loudness of the background noise. The model used to obtain the specific loudness $N_{Target}^{{}^{\prime }}$ for the target sound is the following:

(2) $$\begin{array}{cc} \; & N_{Target}^{{}^{\prime }}=\left\{{\left[\left(E_{Target}+E_{Background}\right)G+A\right]}^{\alpha }-A^{\alpha }\right\}\\ \; & -C\left\{{\left[\left(E_{Background}\left(1+K\right)+E_{THRQ}\right)G+A\right]}^{\alpha }-{\left(E_{THRQ}G+A\right)}^{\alpha }\right\}{\left(\frac{E_{THRB}}{E_{SIG}}\right)}^{0.3} \end{array}$$

where, $E_{Target}$ and $E_{Background}$ are the excitation levels evoked by the target and background sound, respectively. $E_{THRB}$ is the peak excitation value for a masked threshold value due to background noise. $E_{THRQ}$ is the peak excitation value of the absolute threshold. *G* is the gain value amplified for a specific frequency band in the cochlea that is constant up to 500 Hz and increases thereafter. *C*, *A*, α and are constants. Moreover, the specific loudness $N_{Background}^{{}^{\prime }}$ for the background sound is the following:

(3) $$\begin{array}{c} N_{Background}^{{}^{\prime }}=C\left[{\left(E_{Background}G+A\right)}^{\alpha }-A^{\alpha }\right] \end{array}$$

To calculate the loudness of time-varying sounds, the temporal integration of instantaneous loudness was conducted using the previously introduced method [29]. The averaging method for instantaneous loudness is like that of an automatic gain control circuit, which calculates long-term loudness. The maximum long-term loudness was selected as the representative value for the loudness of a nonstationary signal varying with time and was suggested as a reasonable representation by Moore and Glasberg [29].

Binaural inhibition is a concept in which the sound heard in one ear reduces the internal response of the signal in the other ear. To consider binaural inhibition, the value to be reduced was defined as the effective factor and calculated using Equation (4).

(4) $$INH_{IPSI}\left(E\right)=2/\left[1+{\left\{sech\left(S_{CONTRA}{\left(E\right)}_{smoothed}/S_{IPSI}{\left(E\right)}_{smoothed}\right)\right\}}^{P}\right]$$

where $INH_{IPSI}$ is the effective factor for one ear. $S_{CONTRA}$ and $S_{IPSI}$ are the smoothed short-term specific loudness of the contralateral and ipsilateral ears, respectively; and P=1.598. The loudness considering binaural inhibition is the value divided by the effective factor from the loudness.

### 2.2. Research Field

A research area should be selected in advance to model the combined noise annoyance. The conditions for selecting the research area are as follows:

- Residents in the research area should be simultaneously exposed to aircraft and road noises.
- No other noise sources (industrial or railway) should exist in the field for evaluating aircraft and road traffic noise sources.

**Figure 1:**
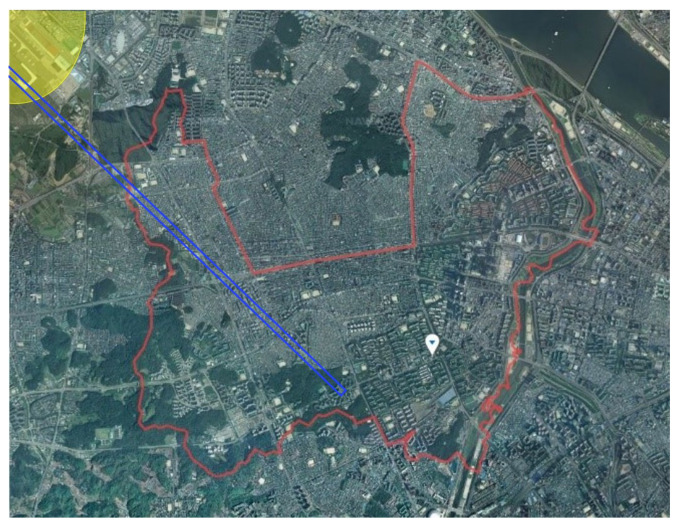
The research area for noise exposure, Yangcheon-Gu (red borderline); Gimpo airport (yellow area); The airway of the airport (blue line).

Considering these conditions, Yangcheon-Gu, Seoul, South Korea was selected as the research field. Yangcheon-Gu is near Gimpo airport and aircraft flyover routes and has a complex road traffic system; thus, residents in the area are exposed to high road traffic noise levels. The research field is shown in Figure 1. The research field is shown in Figure 1. The red border is Yangcheon-gu, the study area, and Gimpo airport is the yellow area in the upper left corner of the figure. The airport route is marked with a blue line.

### 2.3. Stimuli

Aircraft and road traffic noises were recorded as stimuli for the experiment. Aircraft noise was measured around Gimpo Airport, and the flyover noise was recorded below the flight route. Five aircraft noises were recorded, one of which was chosen as the test stimulus. Road noise recordings were conducted on an eight-lane road in Seoul. The recording time was between 15:00 and 15:30 to avoid traffic congestion. The total recording time was 30 min. A normal recording period of 20 s was selected and used as the experimental stimulus.

Head & torso simulator (HATS type 4100, B & K, Nærum, Denmark) was used to record binaural sounds. The HATS has two channels with a microphone in the ear position for binaural recording; pinna-shaped rubber forms and a body-shaped torso duplicate the diffraction of sound by the pinna and the body.

**Figure 2:**
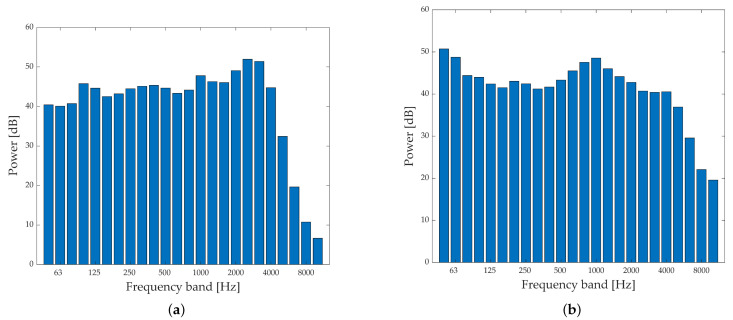
1/3 Octave band of test stimuli (60 dB): (**a**) Aircraft Noise; (**b**) Road Traffic Noise.

The spectral characteristics of the recorded sound used as the experimental stimuli are shown in Figure 2 and displayed in the form of a 1/3 octave band; both sounds are adjusted to 60 dB. Aircraft noise has high-frequency components, and road traffic noise has a broadband property. The recorded sounds were employed in the validation and in the modeling of short-term annoyance.

### 2.4. Validation Test of a Partial Loudness Model with Binaural Inhibition

Before applying a partial loudness model with binaural inhibition, it was necessary to validate whether the appropriate environmental noise assessment was performed.

#### 2.4.1. Stimuli

Aircraft and road noises were selected for the validation test. The levels of aircraft noise were selected considering the practical exposure range of residents near airports. The range of the maximum noise level of the aircraft for one flyover is 70–80 dBA, and when converted to A-weighted equivalent continuous noise levels (LAeq), the range is 68–78 dBA. Therefore, the aircraft sound stimuli were adjusted to 68, 73, and 78 dBA. Road traffic noise in this area was measured to be 55–75 dBA according to the traffic density. As such, levels of 55, 65 and 75 dBA were selected to represent low, medium and high traffic densities. The road traffic sounds were adjusted to 55, 65, and 75 dBA.

#### 2.4.2. Experimental Equipment

The experiment was conducted in an anechoic chamber (3.2 × 3.2 × 2 m^3^) with an ambient sound level of approximately 20 dBA and a cut-off frequency of 200 Hz. The test was conducted with a laptop connected to a digital-to-analog converter and signal data acquisition hardware (LAN-XI, bp2215, B&K, Nærum, Denmark). The DAC executed the sounds from the laptop to the headphone with a flat frequency response (SRH1840, Shure, Chicago, IL, USA), and the LAN-XI calibrated the output signals to adjust the target noise levels. The subjects wore headphones and followed instructions on the laptop screen.

#### 2.4.3. Participants

A total of 14 subjects (3 females and 11 males) were selected for the validation test. Before the primary test, a screening test was conducted to check if the subjects had hearing issues; all subjects had normal hearing. The age range was 22 to 30 years, with an average of 28.7.

#### 2.4.4. Procedure

The experiment was designed to compare the loudness of aircraft noise in quiet conditions and with background noise. The up-down method was adapted for the experiment [33]. In this test, road traffic noise was chosen as the background noise. The validation procedure was as follows:

- The subjects first heard aircraft noise without background noise. Next, the subjects listened to aircraft noise with road traffic noise in the background.
- The pre-programmed computer then asked a comparison of whether the aircraft noise with the background noise was quieter, louder, or equal to the aircraft noise in the quiet environment.
- The program was adjusted the level of the stimuli according to the responses of the subjects. For example, if the subject answered that the aircraft noise was louder with background noise than in quiet conditions, the aircraft noise level with background noise was reduced in the next session.
- The level change steps decreased from 5 dB to 1 dB.
- The experiment proceeded until the subjects select “same”.
- Nine scenarios (three aircraft noises × three road traffic noises) were conducted in combination with each dB of aircraft noise and road noise.

All the experimental procedures were pre-programmed using MATLAB 2019b. Subjects read the instructions, listened to the stimuli, and entered the test results without the assistance from the experimenter. The test lasted approximately 90 min, with three 5-min breaks when the background noise level changed.

### 2.5. Jury Test for Modeling of Short-Term Annoyance

The laboratory experiment was conducted to evaluate the perceived annoyance of the combined noise.

#### 2.5.1. Stimuli

The laboratory test stimuli for annoyance evaluation were the same as in the validation test, except that two stimulus sound levels were added. The aircraft sound stimuli were adjusted to 63, 68, 73, 78, and 83 dBA; and the road traffic noise sounds were adjusted to 55, 60, 65, 70, and 75 dBA.

#### 2.5.2. Experimental Equipment

The experimental equipment used in this test was the same as that used in the previous test.

#### 2.5.3. Participants

Fifty subjects participated in the experiment, including 11 women and 39 men. The age range was 22–32 years, with an average of 25.3 and SD = 3.15. Participants of the experiment participated in the university community and received credit as a reward for participation in the experiment. Before the test (as in the previous test), a screening test was conducted to check for hearing issues; all subjects had normal hearing.

#### 2.5.4. Procedure

The experiment was conducted by responding to the degree of annoyance felt by the experimenter upon hearing the stimulating sound. The answers are given as a score from 0 to 10 on an 11-point numeric scale following ICBEN recommendations [34]. The experimental process was as follows:

- The subjects heard the aircraft noise in quiet conditions and scored the annoyance from the aircraft noise on the 11-length scale.
- Next, they heard the road traffic noise in quiet conditions and scored the annoyance from the road traffic noise on the 11-length scale.
- The aircraft noise was played with the road traffic noise; the subjects were asked to score the level of annoyance of the aircraft noises in the presence of road traffic noise.
- The road traffic noise was played with the aircraft noise, and the experimenter asked the subjects to rate the level of annoyance of the road traffic noise in the presence of aircraft noise on an 11-point numeric scale.
- The combined aircraft and road traffic noises were played, and the subjects were asked to rate the level of annoyance of the total combined noises on the 11-point numeric scale.

Total 85 noises, with five aircraft noises, five road traffic noises, twenty-five combination of aircraft noise with road traffic sounds, twenty-five combined noises of road traffic noise with aircraft sounds, and twenty-five aircraft and road traffic noise combination, were played and the test lasts about 70 min. The level of stimuli was played randomly to minimize the impact on the previous sound source.

### 2.6. Long-Term Annoyance Model

A long-term annoyance model from the combined noise was evoked by residents exposed to combined noise for more than a year. The long-term model was derived using the loudness calculated from the noise map of the research field and the survey data.

#### 2.6.1. Noise Mapping

**Figure 3:**
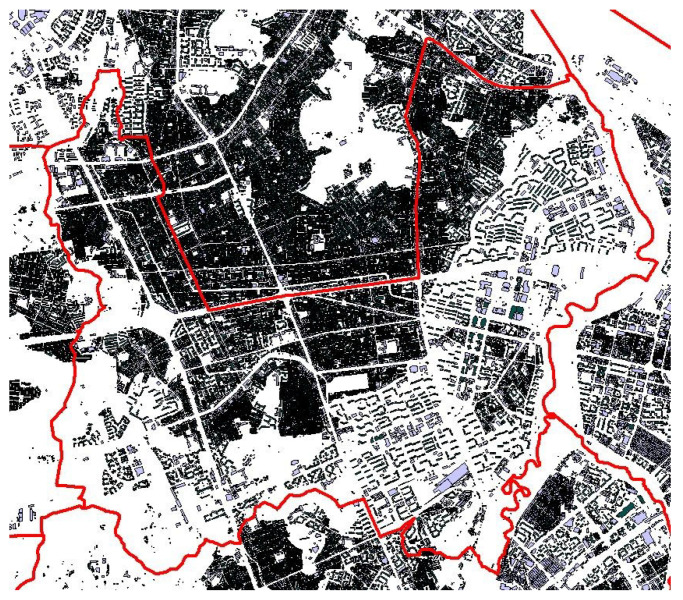
3D geographical data of research field. Buildings of the research area by CadnaA.

The noise map was depicted using the commercial software CadnaA Version 2017 (Datakustik, Munich, Germany) [35]. The research area was modeled using 3D geographic data, including contours, buildings, and roads. The data are shown in Figure 3.

Aircraft noise modeling programs currently in use include ANCON [36], INM [37], IsoBella [38] and STAPES [39]. In this study, aircraft noise from the research area was calculated using the commercial software Integrated Noise Model version 7.0 (INM, Federal Aviation Administration, Washington, DC, USA) [37]. INM is a computer model that evaluate aircraft noise impacts in the vicinity of airports. It uses noise-power-distance (NPD) data to estimate noise accounting for specific operation mode, thrust setting, and source-receiver geometry, acoustic directivity, and other environmental factors.Aircraft noise was predicted using NPD data, runway information at the airport, average daily traffic, and aircraft type. Runway information at the airport, average daily traffic, and type of aircraft were used as inputs. The noise map grid was 10 m × 10 m. The unit of aircraft noise is LAeq, which is the average annual noise level of the research field.

A map of road traffic noise was depicted with measured traffic density and noise levels. To predict the road traffic noise of the research area, road traffic density, the ratio of heavy vehicles, the speed limit of each road, and road materials were evaluated. Measurements of road traffic density were conducted from 11:00–11:30 and 15:00–15:30 (daytime), 20:00–20:30 (evening), and 00:00–00:30 and 03:00–03:30 (nighttime) at 45 points to cover all the roads in the research area to avoid rush hour to measure average traffic density. The data measured in 30 min was doubled to convert to 1 h data. Road traffic density of daytime and nighttime were determined by the average of each of the two measurement results. Measurements were made at 45 points to cover all of the study areas. Noise barriers of road were not included in 3D geographical data, so that they were manually added to each road from CadnaA. The noise prediction model for road traffic is RLS-90, Which is the following equation [35].

(5) $$L_{m,E}=37.3+10log\left[Q\left(1+0.082P\right)\right]$$

(6) $$L_{m}=L_{m,E}+R_{SL}+R_{RS}+R_{RG}+R_{E}+R_{DA}+R_{GA}+R_{TB}$$

(7) $$L_{r}=L_{m}+K$$

The coefficients of road traffic noise model are as follows:

1. $L_{m,E}$: Sound emission level at 25m distance from the source under idealized condition (speed 100 (80) km/h for a light (heavy) vehicle, road gradient < 5%, smooth asphalt)
2. $L_{m}$: Mean emission level for each lane at a receiver position
3. $L_{r}$: Sound level at a receiver position
4. *Q*: Traffic volume per hour
5. *P*: Percentage of heavy trucks P (Weight > 2.8 tons)
6. $R_{SL}$: Correction for the speed limit
  (a) $R_{SL}=L_{car}-37.3+10log\left[\frac{100+(10^{0.1D}-1)P}{100+8.23P}\right]$
  (b) $L_{car}=27.7+10log\left[1+{\left(0.02v_{car}\right)}^{3}\right]$
  (c) $D=L_{truck}-L_{car}$
  (d) $L_{truck}=23.1+12.5log\left(v_{truck}\right)$
  (e) $v_{car}$: the speed limit ranged from 30 to 130 km/h for light vehicles
  (f) $v_{truck}$: the speed limit ranged from 30 to 130 km/h for light vehicles
7. $R_{RS}$: Correction for road surface
8. $R_{RG}$: Correction for road gradient
  (a) $R_{RG}=0.6\left|g\right|-3$ for $\left|g\right|>5\%$
  (b) $R_{RG}=0$ for $\left|g\right|\leq 5\%$
  (c) *g*: Road gradient
9. $R_{E}$: Correction for the absorption characteristics of building surfaces
10. $R_{DA}$: Attenuation coefficient for the distance and air absorption
11. $R_{GA}$: Attenuation coefficient due to ground and atmospheric conditions
12. $R_{TB}$: Attenuation coefficient due to topography and buildings dimensions
13. *K*: Increased effect of light controlled intersections

**Figure 4:**
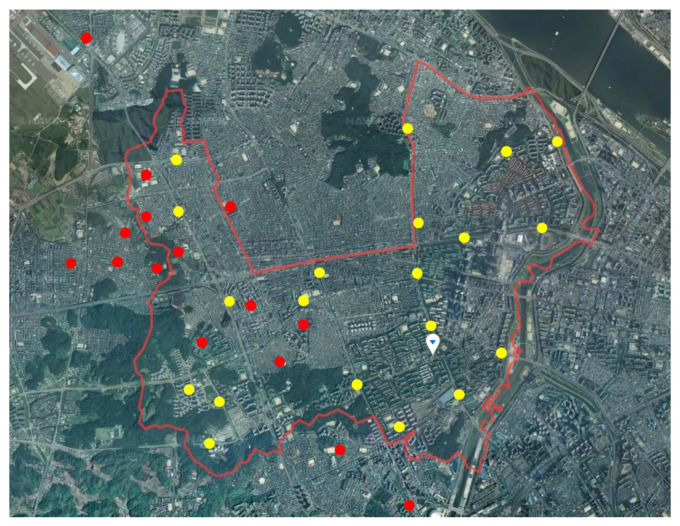
Noise measurement points. Aircraft (red circles); road traffic (yellow circles) noise.

The validation process using the measurement data is essential during the noise map preparation process. To validate the noise maps, we compared the results of noise measurements and predictions in the research field. The aircraft noise levels of 15 points were measured automatically by an airport noise monitoring system. Fifteen points of aircraft noise measurement are shown in a red circle in Figure 4. Road traffic noise measurements to validate the noise map were measured on 20 points, where the points are shown in the yellow circle of Figure 4. The measurement procedure was carried out according to the ISO standard [40]. Noise measurement data were collected by Class 1 sound level meter (type 2250, B & K, Nærum, Denmark) at the height of 1.5 m from the side surface of the road. Measurements were made at the same time as traffic density measurements. Daytime and nighttime noise levels were measured twice and determined as the mean. Based on this, measured day-night average sound levels were compared with predicted levels.

**Figure 5:**
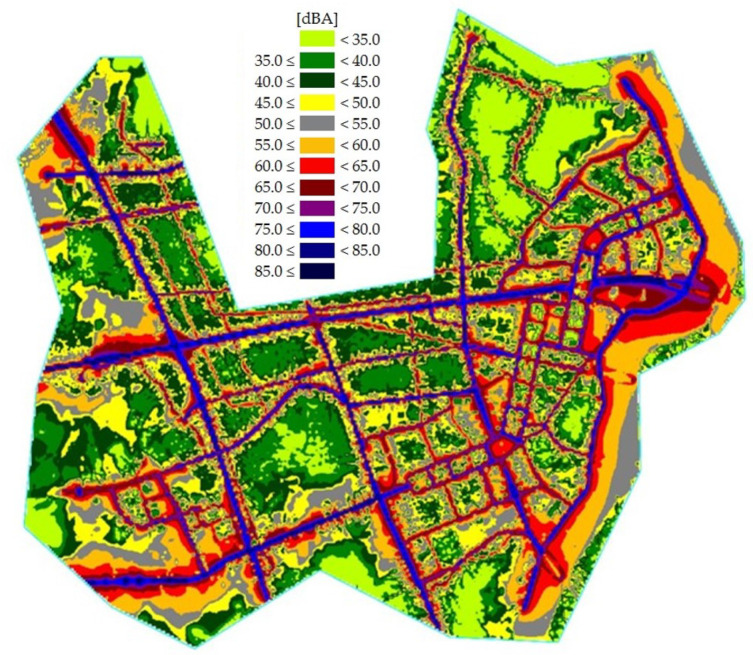
Road traffic noise map of the research area.

**Figure 6:**
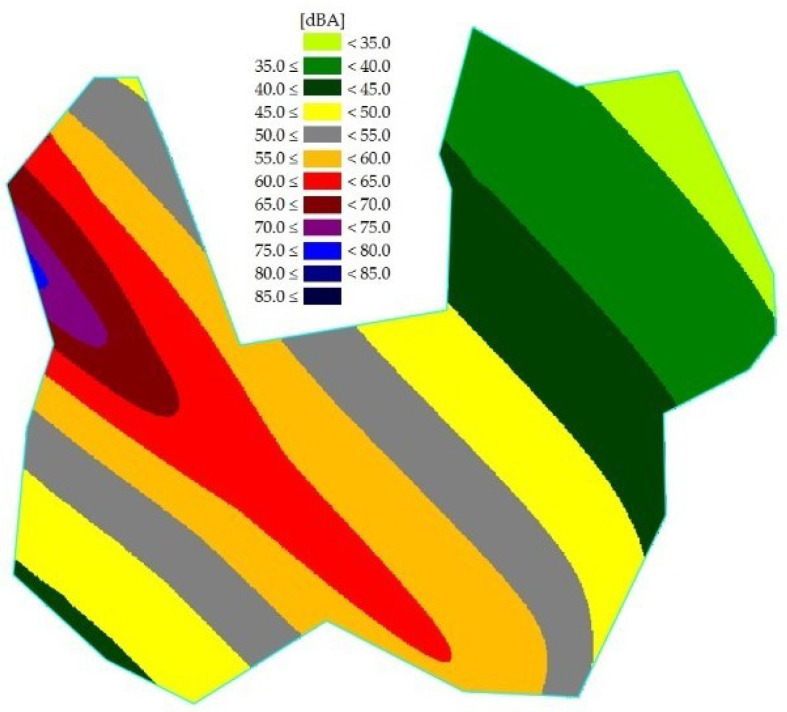
Aircraft noise map of the research area.

**Figure 7:**
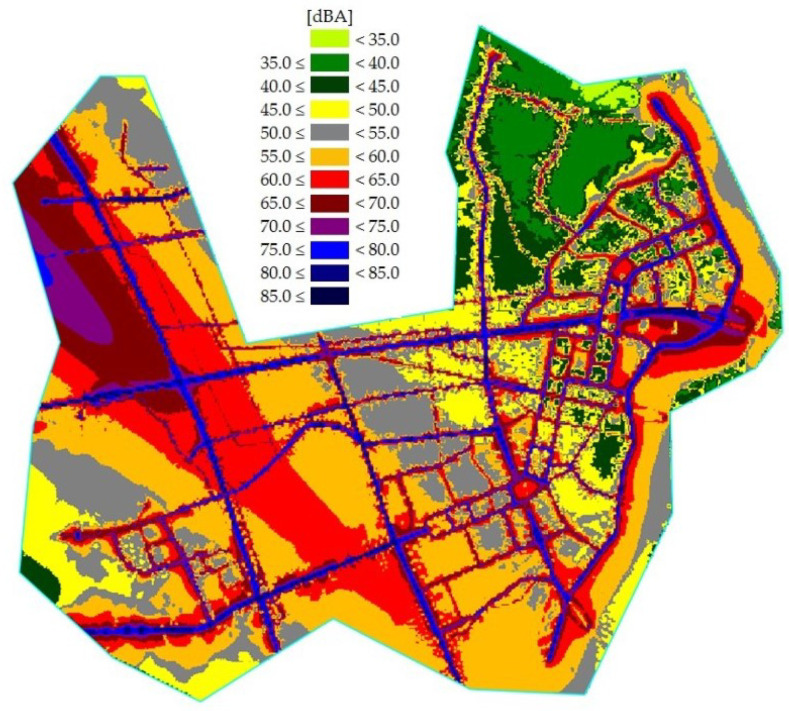
Combined noise map of the research area.

According to the Ordinance of the Ministry of Environment of the Republic of Korea, the validation standard for noise maps is less than 3 dB. Aircraft noise map data have good predictability, with an error of less than 3 dB; and the road traffic noise map had an error of less than 2 dB. In general, it is very difficult to have an error of less than 3 dB due to uncertainty in the prediction of the noise model and measurement. Uncertainty can be reduced by using accurate measurement results. In this research, various types of data were used in the aircraft noise prediction process, and road noise was measured at as many points as possible, so the noise map could be predicted with high accuracy. Validated aircraft and road traffic noise maps are shown in Figure 5 and Figure 6, respectively. In addition, the combined noise map is shown in Figure 7.

#### 2.6.2. Field Survey

To assess the community response to combined noise, we employed a field survey. The survey data conducted in the previous study was used for this study [41]. The depicted noise maps were used to select the survey subjects using the range of noise levels in the research area. For the convenience of selection, the change in noise levels according to height was not considered. The survey subjects were selected based on the noise level of the noise map regardless of the height. 1000 subjects were selected by grouping (Table 1). Table 2 shows the social demographic data of the participants.

The survey was conducted by visiting the residence of the subjects and asking if they could participate in the investigation of aircraft and road traffic noise annoyance. Researchers asked about the annoyance from aircraft noise with road traffic noise (and vice versa) at home. Experimenters answered their annoyance ratings on the 11-length scale with 0 as not annoyed at all and 10 as extremely annoyed, according to ICBEN recommendations [34].

#### 2.6.3. Calculation of Partial Loudness Regarding Points of the Survey Area

The loudness of aircraft and road noises in the residence of each subject was calculated using a partial loudness model. The sound level, which is the input value of the loudness model, was corrected by assuming the indoor noise level of each noise source to which the experimenter is exposed in the residence. The difference between the outdoor noise and the indoor noise depends on whether the window is open or closed. In this study, it is assumed that all subjects’ home windows are slightly open to set to the mean value. In this condition, the corrected values were 15 and 15.8 dBA for aircraft and road traffic noises, respectively [42,43,44,45,46,47].

### 2.7. The Statistical Analysis

In many regression methods, logistic regression is a statistical model with the form of a logistic function, as expressed in Equation (8):

(8) $$f\left(x\right)=\frac{L}{1+e^{-k\left(x-x_{0}\right)}}$$

where $x_{0}$ is the value of Sigmoid’s midpoint; *L* is the maximum value of the curve; and *k* is the logistic growth rate. The logistic function has a maximum limit that is derived by an infinite value of the independent variable. Statistical analysis was performed using SPSS 25.0 software (SPSS Inc., Chicago, IL, USA).

### 2.8. Annoyance Map

The annoyance map of combined noise is depicted using the annoyance model but only shows the total annoyance from each noise source; therefore, partial annoyance—which includes the contribution to the annoyance for each source—is not considered [16,17]. In this study, the background noise effect was considered using a partial loudness model. The relationship between each noise source can be visually observed when applied to the annoyance map.

**Figure 8:**
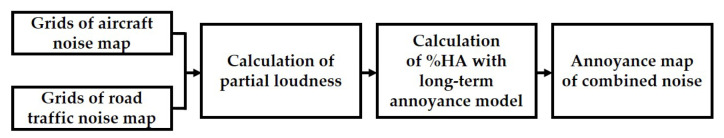
Annoyance mapping procedures.

The annoyance map was constructed using CadnaA in the same manner as the noise map. CadnaA and other noise mapping programs cannot calculate loudness or annoyance. Thus, annoyance data are calculated from the long-term annoyance model, and only the calculated annoyance data are inserted to show the annoyance distribution. First, grids were drawn in units of 10 m × 10 m in the research field. The partial loudness was calculated using the aircraft and road noises corresponding to each grid, and the corresponding %HA (the highly annoyed percentage) was calculated using the long-term annoyance model and expressed as CadnaA. %HA represented the percentage of residents who gave the annoyance a rating of more than eight on the 11-length scale [48]. The whole procedures are depicted as block diagrams in Figure 8.

## 3. Results

### 3.1. Validation of the Partial Loudness Model with Binaural Inhibition

The results of the validation tests are presented in Table 3. According to the experimental results, when comparing the loudness level between noise in quiet and noise with background noise, the subjects hear the same when the noise with background noise is louder. For example, subjects reported that 78 dB of aircraft noise with no background noise and 80.1 dB of aircraft noise with 75 dB of road noise as background noise were heard at the same level.

**Figure 9:**
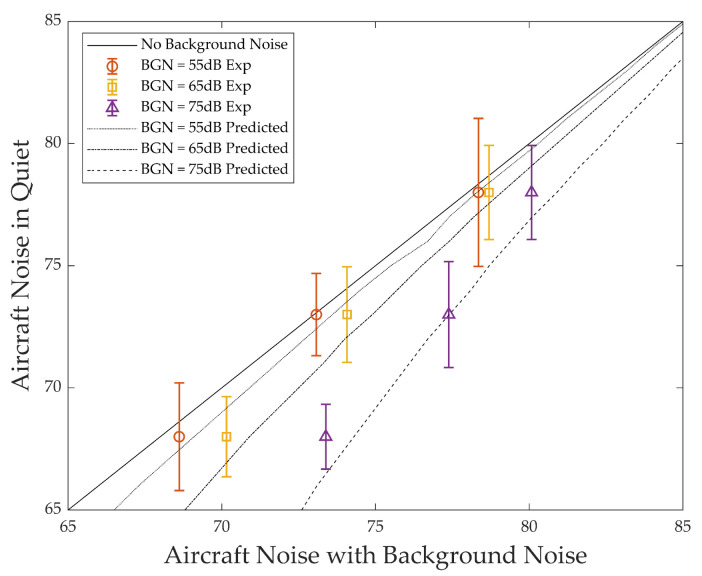
Comparison between experimental results and predicted results.

The results predicted using the partial loudness model were like the experimental results. Further, to compare the tendency of the experimental results and the predicted results, an equal loudness noise curve in the adjacent noise range was created. These curves and the test results are depicted in the same graph to compare the predicted and experimental results. The graph with the standard deviation is depicted in Figure 9.

The horizontal axis of the graph is the level of aircraft noise with road traffic noise, and the vertical axis is the level of aircraft noise in quiet. Each point on the graph refers the level at which both sounds are perceived as the same loudness. BGN on the graph means the background noise level. Each line in the graph is the result of prediction using the partial loudness model. Error bars are the results of Table 3 shown with the standard deviation. Experimental and predict results show that they are consistent in their tendency. The model validation revealed that the calculations of partial loudness by combined noises are executed with the partial loudness model of binaural inhibition.

### 3.2. Perceived Annoyance in Laboratory Tests

**Figure 10:**
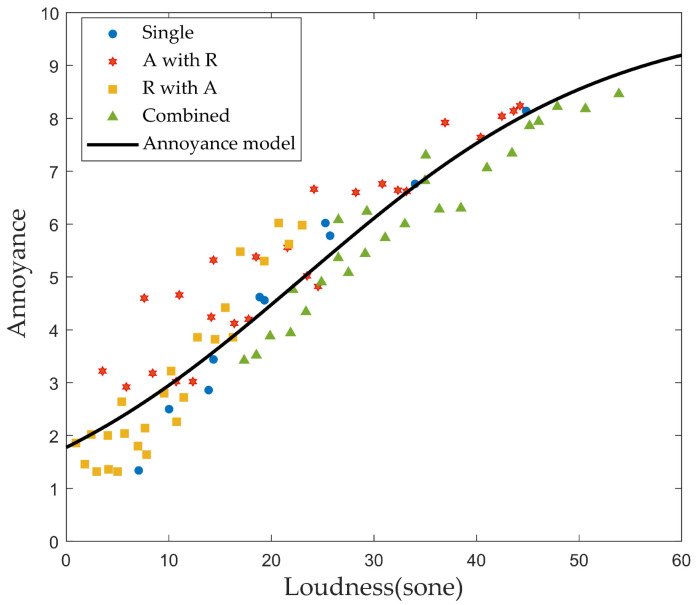
Short-term annoyance model.

The calculation results for short-term annoyance are shown in Figure 10. Single, partial, and total loudness values were calculated according to the experimental situation; and the corresponding annoyance values of the sound source were arranged. The results for each stimulus were classified by a marker. Single is the single noise, indicated by a blue circle. A with R means the aircraft noise with road traffic noise as background noise, indicated by a red hexagonal star. R with A means road traffic noise with aircraft noise as background noise and is marked with a yellow square. Finally, Combined means the combined noise of aircraft and road traffic, indicated by the green triangular marker. Because the loudness units of the experimental sound source were the same, the loudness and the annoyance scores were placed on the horizontal and vertical axes, respectively.

As in previous work, we conducted logistic regression analysis for a 10-point annoyance rating. The annoyance model (Equation (9)) was derived as follows:

(9) $$Annoyance=\frac{1}{0.1+0.463\times 0.936^{Loudness}}$$

The determinant of the short-term annoyance model (0.939) shows that the regression curve follows the test results significantly. Because loudness is the only independent variable, annoyance can be calculated. Additionally, the partial annoyance can also be calculated by calculating the partial loudness of the combined noise.

### 3.3. Perceived Annoyance in Field Survey

The loudness values of aircraft and road noises corresponding to the residence of each subject were calculated using a partial loudness model. Three thousand loudness values were derived up to the partial loudness according to the target noise and the total loudness value of the combined noise. For long-term annoyance, a value of %HA was adopted. To calculate the percentage, the loudness values were grouped into certain ranges. In this study, nine groups were generated with a range of loudness in five sones. Figure 10 shows the relationship among the values of partial loudness and %HA.

**Figure 11:**
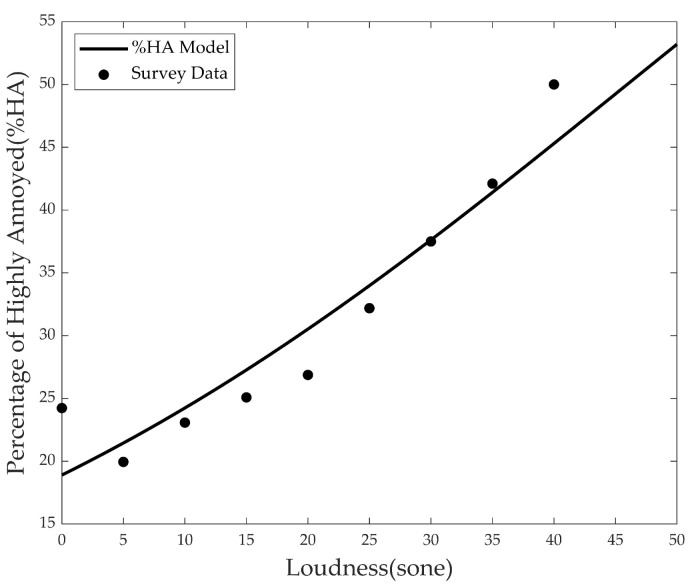
Long-term annoyance model.

According to the survey data in the Figure 11, when the loudness is less than 20 sone, the slope of %HA is also gentle, but when it is greater than 20 sone, the slope in %HA also increases rapidly. Moreover, the result in the group with a loudness of 0 is very interesting. We performed logistic regression analysis using the results of %HA and loudness. Logistic regression analysis was used to model the correlation between loudness values and %HA. The deduced equation is as follows, with an R^2^ value of 0.882:

(10) $$\%HA=\frac{1}{0.01+0.043\times 0.97^{Loudness}}$$

According to the survey data in Figure 10, when the loudness is less than 20 sone, the slope of %HA is also gentle, but when it is greater than 20 sone, the slope in %HA also increases rapidly. Interestingly, it is shown that even in the group with a loudness of 0, there are some highly annoyed populations. We performed logistic regression analysis using the results of %HA and loudness. The regression curve closely follows the investigation result. Equation (8) is the final form of the long-term annoyance model that combines aircraft noise and road traffic noise. If the aircraft and road traffic noise levels are known within the research field, the percentage of highly annoyed residents due to noise can be deduced from the model.

### 3.4. Annoyance Map

**Figure 12:**
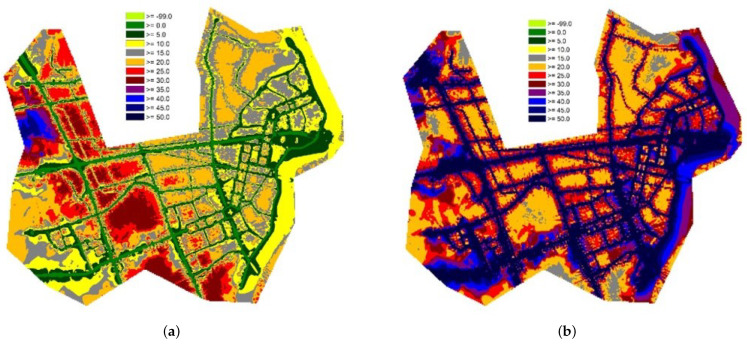
Annoyance maps: (**a**) Aircraft Noise with road traffic noise as background sound; (**b**) Road Traffic Noise with aircraft noise as background sound.

Figure 12a is an annoyance map generated by aircraft noise with road traffic noise (background noise); Figure 12b is an annoyance map generated by road traffic noise with aircraft noise (background noise). Figure 12a shows the highly annoyed distribution at the airport, in the flight path of the aircraft, and in the vicinity.

## 4. Discussion

In this study, we presented a new perspective for the evaluation of annoyance of combined noise. Both intensity and frequency components of noise could be considered using the loudness, and the masking effect between each noise source within the combined noise was quantified using the partial loudness.

### 4.1. Perceived Annoyance in Laboratory Tests

**Figure 13:**
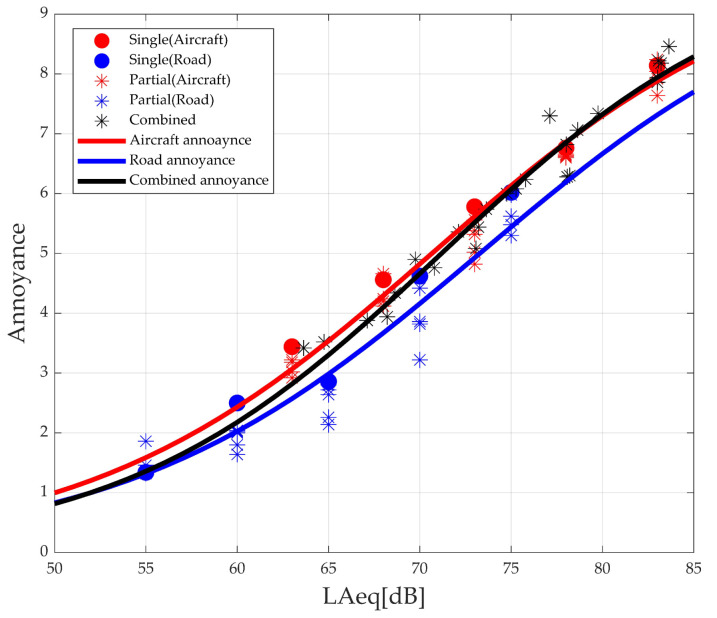
Annoyance evaluation results according to the noise level.

The laboratory tests allowed only acoustic effects of noise to be considered. Figure 13 shows the results of analyzing the experimental results in terms of the noise level. The figure shows that annoyance of aircraft noise is higher than road noise, which was also reported in previous studies [11,12,13]. When only single annoyance values are compared, even when the aircraft noise level is smaller than the road noise level, the response to the annoyance evaluation is the same or higher. Furthermore, comparing the results of single and partial annoyance within the same noise source shows that single annoyance is mostly more immense, demonstrating that the masking effect between noise sources occurs [10]. In this study, the characteristics of each noise source and the masking effect could be integrated into one variable by adopting partial loudness.

### 4.2. Perceived Annoyance in Field Survey

**Figure 14:**
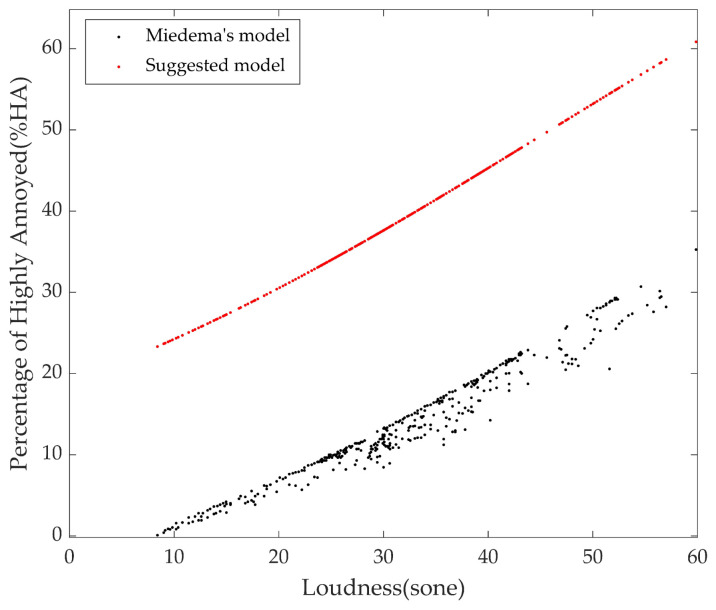
Comparison between the suggested model in this study and Miedema’s annoyance model.

The long-term model derived in this study was compared with Miedema’s model [8]. Because Miedema’s model adopted the equivalent noise level of aircraft and road traffic noises, the noise level was converted to the loudness to compare the two models. The tendencies of the models are similar; but the annoyance value of the model proposed in this study is higher than that of Miedema’s model (Figure 14). The new model includes the total annoyance of combined noise and the partial annoyance of aircraft and road traffic noises. Further, because annoyance is a subjective psychoacoustic value, differences may occur depending on the country, region, or subject. Therefore, we suggest that the model developed in this study is more suitable for subjects residing in Korea.

### 4.3. Relationship between Laboratory and Field Studies

Results from laboratory field studies are of limited comparability due to inherent differences. Laboratory and field studies can be distinguished because the experimental environment and conditions are different. In laboratory studies, subjects experimented in the anechoic chamber, focusing on acoustic characteristics. The types of sound heard by subjects were limited to aircraft noise and road traffic noise. It was an environment where subjects could concentrate on only these two noises. Therefore, it was an ideal condition in terms of acoustic characteristics.

On the other hand, field studies were conducted for residents who have been exposed to noise for more than a year. Participants answer the questionnaire relied on their memory. In addition to noises covered by this study, there are many other noises in society, such as construction and neighborhood noise. Moreover, non-acoustic effect modifiers are always present in the field. For these reasons, it can be seen that the realistic environmental condition is reflected.

In the laboratory study, acoustic effects were enhanced, and non-acoustic effects were excluded. The acoustic effect is relatively weak in the field study, but the non-acoustic effect was also considered. Thus, both two studies are complementary.

### 4.4. Annoyance Map

A peculiar feature on the annoyance map is the relatively small proportion of annoyance near roads. This is because annoyance is applied based on different levels of background noise, even at the same noise level [49]. The noise maps of the previous studies did not show this effect. Figure 12b shows the distribution of annoyance near the road; unlike in Figure 12a, the effect of background (aircraft) noise is almost unnoticeable.

**Figure 15:**
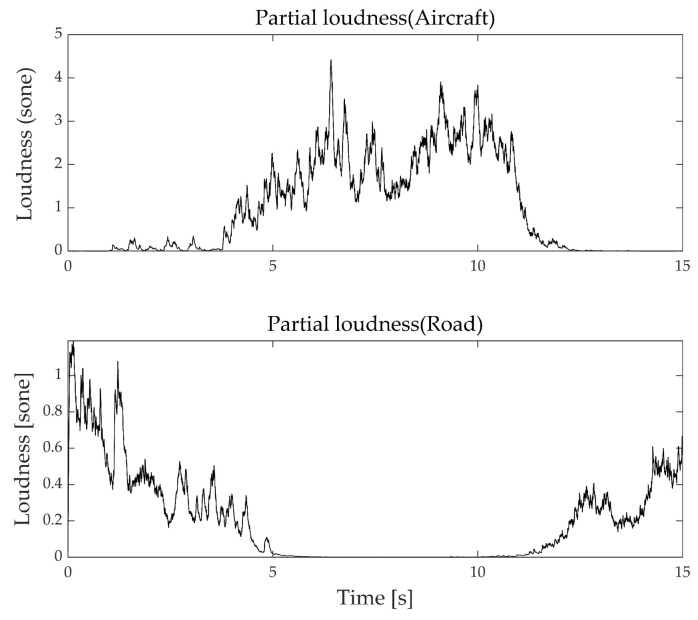
Partial loudness in combined noise.

The difference in the background noise effect of aircraft and road traffic noise can be explained by two main reasons. First, the exposure time and intensity of aircraft and road traffic noises are different. Aircraft noise is characterized by impact, and road noise is characterized by continuity. Therefore, the effects on the background noise are different. Background road noise affects aircraft noise before and after the flight; however, aircraft background noise only affects road noise during the flight. Therefore, subjects tend to perceive road noise as the background rather than aircraft noise. The second reason is the difference in a physical aspect. Figure 15(upper) is partial loudness of aircraft noise when road traffic noise level is 75 dBA and aircraft noise level is 63 dBA. While road traffic noise level is higher than the aircraft noise level, aircraft noise can still be perceived. Figure 15(lower) is partial loudness of road traffic noise when the aircraft noise level is 83 dBA and road traffic noise level is 55 dBA. It is entirely different from Figure 15(upper), partial loudness is measured only at the beginning and end of the road traffic noise playtime. The middle part is completely masked by aircraft noise, and subjects do not recognize the road traffic noise. For these reasons, it causes differences in the annoyance maps of aircraft and road traffic noise.

### 4.5. Limitations

There are some limitations to our study. First, in a laboratory test, the levels of stimulus sounds were adjusted in the amplitude without spectral characteristics, atmospheric absorption, and ground effects. Nevertheless, we speculated that the difference with the actual noise was minimized because noise sources with similar intensity were adjusted. Second, although this study developed the combined noise assessment model with aircraft and road traffic noise, the area to which the model can be applied is limited and specific. Finally, Annoyance models in this study were developed based on the assumption that it was most appropriate to analyze the masking effect. However, since the only variable in models is loudness, this is accompanied by the risk of not fully explaining the annoyance. Other metrics may also be affected by masking effects, but have not yet been clarified. Therefore, this study was conducted considering only partial loudness.

### 4.6. Future Work

Future studies investigating annoyance assessment in different research areas with the same conditions will be helpful to provide information about the scalability of the model developed in this study. In addition to aircraft and road traffic, there are various types of noise, including railway, industrial, and drone noise. It would also be of value to investigate other combinations, which may vary depending on each noise’s intensity and frequency characteristics.

## 5. Conclusions

This study developed annoyance models for combined noise using partial loudness and implemented the annoyance as a visual map. Partial loudness, a psychoacoustic factor, was adopted to consider that annoyance response varies depending on the noise source and that a masking effect occurs between each noise source within the combined noise. A modified model was proposed to calculate the loudness by combining the previous partial loudness model with the binaural suppression of the loudness model and validated through experimentation. Laboratory tests and surveys were conducted to evaluate annoyance due to noise combined with aircraft noise and road traffic noise. These results were developed into a short-term annoyance model and a long-term annoyance model, combined with the modified partial loudness model proposed in this study. In addition to the total annoyance due to the single and the combined noises of aircraft and road traffic noise, the partial annoyance values of the target noise with background noise are also evaluated. Implementing the long-term annoyance model to the noise mapping program deduces the annoyance map of the research area. The annoyance maps show how people are annoyed by the combined noises and the single noise source with the background noise. In this research, the annoyance map of aircraft noise with background noise as road traffic noise and road traffic noise with an ambient sound of road traffic noise are depicted. Annoyance maps show the background noise effect, which is not seen in general noise maps. Moreover, it is shown how aircraft and road traffic noises act as background noise. In the aircraft noise annoyance map, the effect of road traffic noise is noticeable, while in the road traffic noise, the aircraft noise barely affects the road traffic noise. 

## Figures and Tables

**Table 1:** Scope of noise levels and the number of subjects.

Group	Noise Range	Subjects
Group 1	less than 50 dBA	454
Group 2	50–60 dBA	274
Group 3	60–70 dBA	210
Group 4	Over 70 dBA	62
Total		1000

**Table 2:** Social Demographical data of survey subjects.

Contents		<55 dB (n = 580)	55–65 dB (n = 275)	65 dB < (n = 145)	*p*-Value
Age (years)		45.4 ± 16.8	44.6 ± 16.1	46.9 ± 15.6	0.398
Residential period (years)		8.5 ± 8.4	7.9 ± 7.3	7.0 ± 5.9	0.112
BMI (kg/m^2^)		23.8 ± 3.7	23.9 ± 3.7	22.7 ± 3.2	0.405
Sex	Male	266	123	59	0.534
	Female	314	152	86	
Educational level	Secondary Level	239	94	52	0.092
	University Level	331	181	91	
Marriage	No	205	100	44	0.462
	Yes	372	175	100	
Monthly income (1000 KRW)	<3000	309	126	53	<0.001
	≥3000	211	133	81	
Smoking	No	469	250	126	0.001
	Yes	111	25	19	
Drinking	No	314	173	90	0.027
	Yes	266	102	55	
Exercise	No	417	183	101	0.277
	Yes	163	92	44	

*p*-Value was calculated by ANOVA for continuous variable and chi-square test for categorical variable.

**Table 3:** Results of validation test for the partial loudness model with binaural inhibition.

Aircraft Noise (dB)	Road Traffic Noise (dB)	Experimental Results (dB)	Predicted Results (dB)	Gap (dB)
68	55	68.8	69.1	0.5
73		73.1	73.6	0.5
78		78.3	78.3	0
68	65	70.2	70.9	0.7
73		74.1	74.9	0.8
78		78.7	79.1	0.4
68	75	73.4	74.3	0.9
73		77.4	77.4	0.0
78		80.1	80.9	0.8

## Data Availability

The associated data of a statistical nature, can be requested to: worry@snu.ac.kr.

## References

[1] ANSI (2004). ANSI S1.1-1994. American National Standard Acoustical Terminology.

[2] World Health Organization (2011). Burden of Disease from Environmental Noise: Quantification of Healthy Life Years Lost in Europe.

[3] US-EPA (1974). Information on Levels of Environmental Noise Requisite to Protect Public Health and Welfare with an Adequate Margin of Safety.

[4] Schulte-Fortkamp B., Weber R. (1997). Overall annoyance ratings in a multisource environment. INTER-NOISE and NOISE-CON Congress and Conference Proceedings.

[5] Berglund B., Lindvall T., Schwela D.H. (1999). Guidelines for Community Noise.

[6] Berglund B., Hassmen P., Job R.F.S. (1996). Sources and effects of low-frequency noise. J. Acoust. Soc. Am..

[7] Smith A. (1991). A review of the non-auditory effects of noise on health. Work Stress.

[8] Miedema H.M., Oudshoorn C.G. (2001). Annoyance from transportation noise: Relationships with exposure metrics DNL and DENL and their confidence intervals. Environ. Health Perspect..

[9] ANSI (2005). Quantities and Procedures for Description and Measurement of Environmental Sound, Noise Assessment and Prediction of Long-Term Community Response.

[10] Brink M., Lercher P. (2007). The effects of noise from combined traffic sources on annoyance: The interaction between aircraft and road traffic noise. INTER-NOISE and NOISE-CON Congress and Conference Proceedings.

[11] Hong J., Kim J., Kim K., Jo Y., Lee S. (2009). Annoyance caused by single and combined noise exposure from air craft and road traffic. J. Temporal Des. Arch. Environ.

[12] Wothge J., Belke C., Möhler U., Guski R., Schreckenberg D. (2017). The combined effects of aircraft and road traffic noise and aircraft and railway noise on noise annoyance—An analysis in the context of the joint research initiative NORAH. Int. J. Environ. Res. Public Health.

[13] Lechner C., Schnaiter D., Bose-O’Reilly S. (2019). Combined Effects of Aircraft, Rail, and Road Traffic Noise on Total Noise Annoyance—A Cross-Sectional Study in Innsbruck. Int. J. Environ. Res. Public Health.

[14] Miedema H.M. (2004). Relationship between exposure to multiple noise sources and noise annoyance. J. Acoust. Soc. Am..

[15] Vos J. (1992). Annoyance caused by simultaneous impulse, road-traffic, and aircraft sounds: A quantitative model. J. Acoust. Soc. Am..

[16] Knauss D. (2002). Noise mapping and annoyance. Noise Health.

[17] Miedema H.M.E. (1992). Response Functions for Environmental Noise in Residential Areas.

[18] Martin M.A., Tarrero A., González J., Machimbarrena M. (2006). Exposure-effect relationships between road traffic noise annoyance and noise cost valuations in Valladolid, Spain. Appl. Acoust..

[19] Birk M., Ivina O., Von Klot S., Babisch W., Heinrich J. (2011). Road traffic noise: Self-reported noise annoyance versus GIS modelled road traffic noise exposure. J. Environ. Monit..

[20] Stoter J., De Kluijver H., Kurakula V. (2008). 3D noise mapping in urban areas. Int. J. Geogr. Inf. Sci..

[21] Law C.w., Lee C.k., Lui A.S.w., Yeung M.K.l., Lam K.c. (2011). Advancement of three-dimensional noise mapping in Hong Kong. Appl. Acoust..

[22] Berger M., Bill R. (2019). Combining VR visualization and sonification for immersive exploration of urban noise standards. Multimodal Technol. Interact..

[23] Scharf B. (1978). Loudness. Handbook of Perception.

[24] Scharf B. (1971). Fundamentals of auditory masking. Audiology.

[25] Moore B.C., Glasberg B.R., Baer T. (1997). A model for the prediction of thresholds, loudness, and partial loudness. AES J. Audio Eng. Soc..

[26] Chun C., Gwak D.Y., Yoon K., Lee S. (2018). Short-term annoyance model of combined aircraft and road traffic noise based on partial loudness model. J. Mech. Sci. Technol..

[27] Kim J., Lim C., Hong J., Jung W., Lee S. (2007). The influence of binaural effects on annoyance for transportation noise. Noise Control. Eng. J..

[28] Glasberg B.R., Moore B.C.J. (2002). A model of loudness applicable to time-varying sounds. J. Audio Eng. Soc..

[29] Moore B.C., Glasberg B.R., Varathanathan A., Schlittenlacher J. (2016). A Loudness Model for Time-Varying Sounds Incorporating Binaural Inhibition. Trends Hear..

[30] ISO 532-1:2017(en) (2017). Acoustics–Methods for Calculating Loudness—Part 1: Zwicker Method.

[31] ISO 532-2:2017(en) (2017). Acoustics-Methods for Calculating Loudness—Part 2: Moore-Glasberg Method.

[32] Glasberg B.R., Moore B.C. (2005). Development and evaluation of a model for predicting the audibility of time-varying sounds in the presence of background sounds. J. Audio Eng. Soc..

[33] Levitt H. (1971). Transformed up-down methods in psychoacoustics. J. Acoust. Soc. Am..

[34] Fields J.M., De Jong R.G., Gjestland T., Flindell I.H., Job R.F., Kurra S., Lercher P., Vallet M., Yano T., Guski R. (2001). Standardized general-purpose noise reaction questions for community noise surveys: Research and a recommendation. J. Sound Vib..

[35] Datakustik, GmbH (2017). Brief Instruction for the Program CadnaA-Software for Noise Abatement. http://www.datakustik.de/.

[36] Ollerhead J., Rhodes D., Viinikainen M., Monkman D., Woodley A. (1999). The UK Civil Aircraft Noise Contour Model ANCON: Improvements in Version 2 (R & D REPORT 9842). Transport.

[37] Boeker E.R., Dinges E., He B., Fleming G., Roof C.J., Gerbi P.J., Rapoza A.S., Hermann J. (2008). Integrated Noise Model (INM) Version 7.0 Technical Manual.

[38] Zaporozhets O. (2016). Aircraft Noise Models for Assessment of Noise around Airports—Improvements and Limitations. ICAO Environmental Report.

[39] STAPES (2009). System for AirPort noise Exposure Studies.

[40] ISO 1996-1:2016 (2016). Acoustics—Description, Measurement and Assessment of Environmental Noise—Part 1: Basic Quantities and Assessment Procedures.

[41] Sung J.H., Lee J., Jeong K.S., Lee S., Lee C., Jo M.W., Sim C.S. (2017). Influence of transportation noise and noise sensitivity on annoyance: A cross-sectional study in South Korea. Int. J. Environ. Res. Public Health.

[42] Jansen G., Linnemeier A., NITZCHE M. (1995). Methodenkritische Uberlagungen und Empfehlungen zur Bewertung von Nachtfluglärm. Zeitschrift für Lärmbekämpfung.

[43] Horne J., Pankhurs F., Reyner L., Hume K., Diamond I. (1994). A field study of sleep disturbance: Effects of aircraft noise and other factors on 5742 nights of actimetrically monitored sleep in a large subject sample. Sleep.

[44] Scheuch K., Griefahn B., Jansen G., Spreng M. (2003). Evaluation criteria for aircraft noise. Rev. Environ. Health.

[45] Hurtley C. (2009). Night Noise Guidelines for Europe.

[46] BUWAL (1998). Belastungsgrenzwerte Für den Lärm der Landesflughäfen. Schriftenreihe Umwelt Nr. 296. https://www.bafu.admin.ch/bafu/de/home/themen/laerm/publikationen-studien/publikationen/belastungsgrenzwerte-laerm-landesflughaefen.html.

[47] Locher B., Piquerez A., Habermacher M., Ragettli M., Röösli M., Brink M., Cajochen C., Vienneau D., Foraster M., Müller U. (2018). Differences between outdoor and indoor sound levels for open, tilted, and closed windows. Int. J. Environ. Res. Public Health.

[48] Miedema H.M., Vos H. (1998). Exposure-response relationships for transportation noise. J. Acoust. Soc. Am..

[49] Lim C., Kim J., Hong J., Lee S. (2008). Effect of background noise levels on community annoyance from aircraft noise. J. Acoust. Soc. Am..

